# Cardiovascular Safety Assessment in Cancer Drug Development

**DOI:** 10.1161/JAHA.121.024033

**Published:** 2021-12-16

**Authors:** Ohad Oren, Tomas G. Neilan, Michael G. Fradley, Deepak L. Bhatt

**Affiliations:** ^1^ Division of Cardiology Massachusetts General Hospital and Harvard Medical School Boston MA; ^2^ Cardio‐Oncology Program Division of Cardiology Massachusetts General Hospital and Harvard Medical School Boston MA; ^3^ Cardio‐Oncology Center of Excellence Division of Cardiology Department of Medicine Perelman School of Medicine at the University of Pennsylvania Philadelphia PA; ^4^ Brigham and Women’s Hospital Heart & Vascular Center and Harvard Medical School Boston MA

**Keywords:** antineoplastic agents, cardiotoxicity, cardiovascular safety, consensus, randomized controlled trials, Quality and Outcomes, Mortality/Survival, Cardio-Oncology

## Abstract

The development of cardiovascular toxicity attributable to anticancer drugs is a pivotal event that is associated with cardiovascular morbidity as well as with worse cancer‐specific and overall outcomes. Although broad consensus exists regarding the importance of cardiovascular safety assessment in cancer drug development, real‐world data suggest that cardiovascular events are significantly underestimated in oncology trials. This drug safety discrepancy has profound implications on drug development decisions, risk‐benefit evaluation, formulation of surveillance and prevention protocols, and survivorship. In this article, we review the contemporary cardiovascular safety evaluation of new pharmaceuticals in hematology and oncology, spanning from in vitro pharmacodynamic testing to randomized clinical trials. We argue that cardiovascular safety assessment of anticancer drugs should be reformed and propose practical strategies, including development and validation of preclinical assays, expansion of oncology trial eligibility, incorporation of cardiovascular end points in early‐phase studies, and design of longitudinal multi‐institutional cardiotoxicity registries.

**Nonstandard Abbreviations and Acronyms**
FDA: Food and Drug Administration
hERG: human ether‐à‐go‐go–related gene
hiPSC: human induced pluripotent stem cell
QTc: corrected QT

Although broad consensus exists regarding the importance of cardiotoxicity assessment in cancer drug development, evidence suggests that cardiovascular events are significantly underestimated in oncology trials.^1^ For example, immune checkpoint inhibitors,^2^ novel anticancer therapy, were initially shown to have no significant cardiovascular sequela in numerous seminal trials.^3^, ^4^, ^5^, ^6^ Nevertheless, pharmacovigilance analyses demonstrated a potentially fatal drug‐mediated myocarditis in 0.06% to 0.27% of patients,^7^ and real‐world data suggest an incidence that is at least 4‐fold higher at close to 1%.^8^, ^9^ Similarly, sunitinib, a multikinase inhibitor, was associated with a favorable cardiovascular safety profile in a phase 3 trial of patients with gastrointestinal stromal tumors^10^ and in those with pancreatic neuroendocrine tumors,^11^ leading to US Food and Drug Administration (FDA) approvals. Additional clinical investigations in patients with metastatic renal cell carcinoma showed that left ventricular dysfunction developed in ≈10% of patients.^12^ As exemplified by these and other anticancer therapeutics, robust characterization of drug‐specific cardiotoxicity rarely emerges in the course of individual clinical trials, which may expose patients to untoward complications. Such drug safety discrepancies have profound implications on drug development decisions, synthesis of risks versus benefits, planning of cardiac surveillance, formulation of cardiovascular prevention strategies, and survivorship.

In this article, we review the contemporary cardiovascular safety assessment of new pharmaceuticals in hematology and oncology, spanning from in vitro pharmacodynamic testing to randomized clinical trials and multi‐institutional registries. We discuss key principles in modern preclinical and clinical investigation, address the challenges intrinsic to effective cardiovascular safety assessment, and propose a road map for the standardized and data‐driven assessment of the biologic and clinical cardiovascular ramifications of anticancer compounds.

## Preclinical Evaluation of Anticancer Drug Candidates

Preclinical cardiac safety testing provides an early signal of a potential cardiotoxic hazard. In addition, it helps characterize specific risks and generate data about mechanisms of cardiotoxicity. Preclinical assays also serve as an important platform for assessment of cardiovascular risk mitigation by specific interventions.

**Figure 1:**
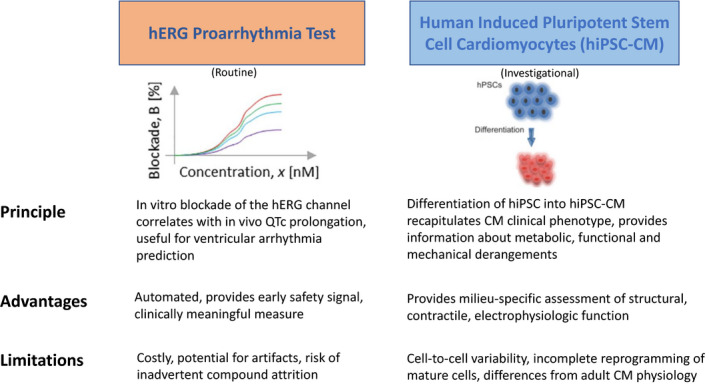
Preclinical tools in the assessment of cardiotoxicity mediated by anticancer drug candidates. CM indicates cardiomyocytes; hERG, human ether‐a‐go‐go–related Gene; hiPSC, human induced pluripotent stem cells; and hiPSC‐CM, human induced pluripotent stem cell–derived cardiomyocytes.

An example of an accepted preclinical assay is the human ether‐à‐go‐go–related gene (hERG) proarrhythmia test, a specialized in vitro study routinely performed in cancer drug development (Figure 1). The assay takes advantage of the hERG potassium channel, which is responsible for the repolarization of the cardiac action potential.^13^, ^14^ Specifically, in vitro blockade of the hERG channel lengthens the ventricular action potential and correlates with electrocardiographic corrected QT (QTc) interval prolongation and ventricular arrhythmias.^15^ The hERG channel inhibition test uses patch clamp electrophysiology, and recent automation of the technique has increased its throughput considerably. The hERG assay has helped identify >100 drugs as potential mediators of long QT syndrome and led to the withdrawal of 10 drugs from the US market between 1997 and 2006.^16^, ^17^ The assay is considered a prerequisite to drug registration, and documentation of minimal hERG liability is needed for a drug to proceed with further testing.^18^ However, several factors affect the utility of this assay. First, although the relationship between hERG blockade and proarrhythmic risk has been established for most noncardiovascular medications, it is more complex and less predictable with anticancer compounds, most notably tyrosine kinase inhibitors. For example, inhibition of hERG potassium currents by nilotinib occurs in therapeutic concentrations and accounts for its clinical QTc prolongation.^19^ In contrast, dasatinib has a maximal inhibitory concentration that is 100 times higher than its estimated maximal concentration, and yet QTc interval prolongation occurs in 1% of patients.^19^ The variability and inconsistencies encountered with the hERG assay stem from the choice of experimental conditions (ie, cell type, temperature), which influence the assessment of channel blockade. In addition, adsorption of hydrophobic compounds to the patch may lead to spurious results, which could be mitigated by the use of glass‐coated compound plates. In addition, the test is time consuming and associated with high costs. These procedural and methodologic caveats highlight the value of instrument and technique standardization as part of the International Council for Harmonization S7B guidelines as well as the need for novel approaches for preclinical arrhythmia assessment.

Human‐induced pluripotent stem cells (hiPSCs) provide valuable insights about drug‐associated cardiotoxicity and are likely to become an integral part of cardiovascular drug assessment in the foreseeable future (Figure 1). At the heart of the technology, hiPSCs are differentiated into cardiomyocytes, and their subsequent maturation and assessment provides important information regarding metabolic, functional, and mechanical derangements.^20^ In addition to standardized protocols for evaluation of cardiac liabilities using hiPSC‐derived cardiomyocytes, newer constructs of varying complexity assess drug influence on the larger cardiovascular system.^21^, ^22^ For example, oncology drugs often lead to multiple cardiovascular pathologies, including atherosclerosis, systemic hypertension, and pulmonary artery hypertension, with one toxicity frequency exerting maladaptive influences on another. Such indirect effects are unlikely to be captured by single‐cell hiPSC‐derived cardiomyocyte assays, which fail to provide data on hemodynamic and neurohumoral factors. To address that challenge, hiPSCs are mixed with fibroblasts, endothelial cells, and mesenchymal stem cells to form 2‐dimensional cultures or 3‐dimensional organoids, which allow more granular evaluation of electrophysiologic abnormalities, structural toxicities, and contractile dysfunction.^21^

HiPSC‐derived cardiomyocytes have also been combined with “omics” studies to identify activated pathways that result in cardiac injury. In one study, doxorubicin triggered apoptotic signaling cascades and upregulated synthesis of proteins (eg, TP53I3, BAG3) that respond to toxic events.^23^ Doxorubicin also led to dysregulation of AMP‐activated protein kinase pathways, which have critical function in p53‐dependent DNA damage and programmed cell death.^23^ In a study of primary cardiomyocyte cell lines, exposure to tyrosine kinase inhibitors correlated with a p26‐gene expression signature and effectively predicted clinical cardiotoxicity.^24^ Further, protein‐protein interaction analyses helped delineate individual kinases and transcription factors that may explain the signature and the associated cardiotoxicity.

Importantly, cancer therapeutic–mediated cardiotoxicity is not always an unpredictable event, and there are genomic markers that can help determine a patient’s likelihood of experiencing toxicity. In that regard, preclinical studies have significantly improved the understanding of the genetic basis of cardiotoxicity. For example, genome‐wide association studies have identified numerous cardiotoxicity loci associated with anticancer drugs (ie, rs28714259 with doxorubicin, *CBR3* and *ABCB1* with trastuzumab),^25^, ^26^, ^27^ and hiPSC models have shed further light regarding plausible genomic biomarkers that predict cardiotoxicity.^28^ This knowledge that there exists a rare population that is genetically predisposed to cardiotoxicity has an impact on safety assessment during drug development and indicates that clinical trials may need to be much larger.

## Cardiotoxicity Assessment in Oncology Trials and Role of Imaging

Although clinical trials serve as an essential vehicle for ascertainment of safety events,^29^ there are no standardized protocols that define best practices for assessment of cancer therapeutic–related cardiotoxicity. Instead, the nature and frequency of monitoring studies are selected according to in vitro cardiac signals, toxicology studies in animals, and pharmacologic class toxicities. With the safety premise of human trials to support reliable detection and characterization of drug‐related cardiac safety events, methods used to screen for cardiotoxicity are ideally highly reproducible and subject to unbiased evaluation by highly experienced personnel. Since no single imaging cutoff value discriminates abnormal from normal, the emphasis should be on developing accurate sequential imaging studies with low test‐retest variance. In addition, safety evaluation instruments need to be readily accessible and reasonably inexpensive to enable implementation in multicenter clinical trials and in real‐world settings.

Assessment of heart failure is a fundamental safety component of clinical trials in oncology. In a contemporary cohort of 2625 heterogenous cancer patients (51% breast cancer) who received anthracycline‐containing regimens, 9% developed cardiotoxicity, defined as left ventricular ejection fraction (LVEF) <50% with >10% absolute reduction from baseline.^30^ A strong relationship between the cumulative anthracycline dose and incident cardiomyopathy was demonstrated, with 3% to 5% developing toxicity with 400 mg/m^2^ and 18% to 48% at 700 mg/m^2^ of doxorubicin.^31^ In a contemporary study of 648 patients with non‐Hodgkin lymphoma treated with rituximab, cyclophosphamide, doxorubicin, vincristine, and prednisone, 29% developed cardiotoxicity, defined as 10% decline in LVEF.^32^ The use of nonpegylated liposomal doxorubicin has been associated with a lower rate of cardiotoxicity relative to that of doxorubicin in several randomized clinical trials of patients with high‐grade lymphoma and breast cancer and is often the preferred approach in patients with left ventricular dysfunction.^33^, ^34^ Trastuzumab, a humanized monoclonal antibody to human epidermal growth factor receptor 2, has also been associated with cardiac dysfunction, albeit with a unique pattern of myocyte injury/dysfunction that is thought to be generally reversible. In a cluster of breast cancer trials, the incidence of cardiomyopathy with trastuzumab ranged from 1% to 28%, and rates were significantly higher when the drug was concurrently administered with anthracyclines. As a result, sequential rather than concurrent administration of trastuzumab and anthracyclines is the standard approach.^35^ Osimertinib, an epidermal growth factor receptor inhibitor used in lung cancer, and sunitinib, a tyrosine kinase inhibitor used in renal cell carcinoma and neuroendocrine malignancies, may also lead to overt heart failure, and monitoring with echocardiography is recommended, although its frequency has not been defined.

Echocardiography remains the most frequently used imaging modality for assessment of ventricular function in clinical trials. Numerous definitions have been used for cardiac dysfunction caused by anticancer drugs (Table).^36^, ^37^, ^38^, ^39^, ^40^ When obtained and read locally, variabilities in echocardiographic measurements and analyses are an inevitable consequence of measurement and process heterogeneity. The echocardiography core lab concept was therefore created to facilitate high‐quality performance, interpretation, and quantification of echocardiograms.^41^ The echocardiography core lab standardizes the imaging process by training sonographers and readers and maintaining adequate intra‐ and interobserver agreement.^41^ The benefits afforded by centralized reading were captured in a prospective imaging trial of patients with stable chest pain in which echocardiography core lab interpretation of coronary computed tomographic angiography led to 41% fewer patients classified as having significant coronary artery disease compared with site interpretation of the same images.^42^ In addition, echocardiography core lab interpretation was demonstrated to have higher accuracy, specificity, and positive predictive value than local site reads.^42^

**Table 1:** Definitions of Cancer Therapy‐Related Cardiotoxicity by Different Cardiovascular and Oncology Societies and Research Groups

Society/Organization	Definition	Anticancer drug	Imaging modality
The American Society of Echocardiography and the European Association of Cardiovascular Imaging^36^	Decrease in the left ventricular ejection fraction >10%, to a value <53%, confirmed with repeat imaging 2–3 wk later	Any	Echocardiography, cardiac magnetic resonance imaging, multigated radionuclide angiography (MUGA)
Food and Drug Administration^37^	Decrease in the left ventricular ejection fraction ≥10% to below the lower limit of normal (or absolute left ventricular ejection fraction ≤45%), or ≥20% absolute decline	Doxorubicin	MUGA or echocardiography
National Cancer Institute^38^	Common Terminology Criteria for Adverse Events: Grade 1: asymptomatic elevation in biomarkers or abnormalities on imaging Grade 2: symptoms with mild exertion Grade 3: symptoms with moderate exertion Grade 4: severe, life‐threatening symptoms requiring hemodynamic support Grade 5: death	Any	Not specified
Breast Cancer International Research Group^39^	Relative reduction in left ventricular ejection fraction >10% from baseline	Trastuzumab and doxorubicin	MUGA or echocardiography
Herceptin Adjuvant Trial Study Team^40^	Reduction in left ventricular ejection fraction ≥10% to <50% at any time	Trastuzumab	MUGA or echocardiography
Cardiac Review and Evaluation Committee^35^	Reduction in left ventricular ejection fraction ≥5% to <55% when accompanies by heart failure signs and symptoms, or a decrease ≥10% to <55% without signs and symptoms	Trastuzumab and doxorubicin	MUGA or echocardiography

Cardiac arrhythmias in patients with cancer are complex disorders that are associated with a significant risk of systemic thromboembolism and cerebrovascular ischemia. Monitoring for arrhythmic disturbances in oncology trials is typically performed using clinical pulse‐based and ECG assessments whose frequency depends on the investigated drug, its pharmacologic properties, and concerning safety signals emerging from earlier‐phase studies. To help address the question of electrocardiographic testing intensity in drug development investigations, the ongoing “thorough QT/QTc study” will help determine whether drugs have a threshold pharmacologic effect on cardiac repolarization (QTc interval increment exceeding 10 ms).^43^, ^44^ The results of this investigation, however, would not be generalizable to patients with cancer, who are more likely to have electrolyte derangements and drug‐drug interactions, and in whom larger mean increases in QTc (ie, >20 ms) are often allowed, accommodating proarrhythmic risks and clinical benefits. Future studies should specifically address the question of ECG frequency in patients with cancer as well as the role of triplicate, 12‐lead ECG monitoring, which has been incorporated into many studies yet lacks high‐quality prospective data to inform its superiority over single ECGs. Finally, whether routine Holter monitoring or more sensitive methods of continuous monitoring using disposable electrode patches or implantable loop recorders improves arrhythmia detection or clinical outcomes in patients with cancer should be evaluated.

## Role of Circulating Biomarkers as Indicators of Cardiotoxicity

Patients with cancer who develop an acute coronary syndrome^45^ or heart failure^46^ have worse clinical outcomes than those without cancer, and circulating cardiac biomarkers represent an important tool for cardiovascular risk stratification and diagnosis.^47^, ^48^ N‐terminal pro‐B‐type natriuretic peptide, a peptide released from the ventricles in response to elevated intracardiac pressures, is an established biomarker in the diagnosis of heart failure.^49^ N‐terminal pro‐B‐type natriuretic peptide levels have been shown to predict ventricular dysfunction and heart failure in patients with cancer receiving chemotherapy or targeted therapies. In a prospective study of 138 women with human epidermal growth factor receptor 2–positive breast cancer who were treated with trastuzumab, N‐terminal pro‐B‐type natriuretic peptide levels at baseline and during follow‐up were associated with development of cardiotoxicity, defined as LVEF <45% or an absolute decline in LVEF >10% or the occurrence of a clinical cardiac event.^50^ Nevertheless, the absolute change in N‐terminal pro‐B‐type natriuretic peptide in that study was small and did not effectively distinguish between patients who developed cardiotoxicity and those who did not.^50^

A prospective investigation of 333 patients with breast cancer who received treatment with anthracyclines demonstrated that brain natriuretic peptide levels >100 pg/mL and LVEF <50% correlated with development of heart failure.^51^ Interestingly, elevated brain natriuretic peptide but not LVEF was predictive of overall death.^51^ These results conflict with those of several studies that revealed no association between natriuretic peptide levels and cardiac dysfunction in patients receiving anthracyclines.^52^, ^53^, ^54^ This inconsistency might be explained by small study sizes, low event rates, patient heterogeneity (different tumor stages, drug regimens, baseline cardiovascular health), variations in laboratory methods, and differences in follow‐up durations.

Cardiac troponins have been shown to be useful adjuncts in the assessment of anthracycline‐ and trastuzumab‐mediated cardiotoxicity. In a prospective evaluation of 205 children with high‐risk acute lymphoblastic leukemia who received doxorubicin, increased pretreatment troponin T levels were associated with abnormally reduced left ventricular mass and end‐diastolic posterior wall thickness at 4 years.^55^ In a cohort of 81 women with human epidermal growth factor receptor 2–positive breast cancer who were treated with anthracyclines followed by trastuzumab, 26 patients (32%) developed cardiotoxicity, as defined by the Cardiac Review and Evaluation Committee,^35^ and ultrasensitive troponin I, measured following completion of anthracycline use, predicted the development of cardiotoxicity.^52^

Whether elevated cardiac troponin levels identify high‐risk cancer patients who might benefit from early institution of cardioprotective measures has not been answered. However, some insights can be derived from a prospective multicenter study of 273 patients with cancer (76% breast cancer) who were randomized into routine enalapril (prevention group) or enalapril only if the troponin was elevated during chemotherapy (troponin‐triggered group).^56^ Patients were treated with a median cumulative doxorubicin dose of 240 mg/m^2^ or epirubicin 360 mg/m^2^. At 12‐month follow‐up, there were no significant between‐arm differences in the incidence of cardiotoxicity, defined as LVEF <50% and representing >10% reduction from baseline.^56^ In a randomized placebo‐controlled double‐blind trial of 130 women with early‐stage breast cancer treated with anthracycline‐containing chemotherapy regimens, candesartan was associated with a lower rate of LVEF decline (0.8% versus 2.6%; *P*=0.026), whereas metoprolol attenuated the increase in cardiac troponins, compared with placebo, although the study population was characterized by an overall low cardiovascular risk.^57^ At 23 months of follow‐up, neither drug protected from LVEF reduction, although candesartan was associated with lower left ventricular end‐diastolic volume and milder declines in global longitudinal strain.^58^

## Cardiovascular Safety Evaluation During Postmarketing Surveillance

Since premarketing trials explore the effects of drugs in relatively small, narrowly defined populations with a typically favorable cardiovascular risk profile, and over a relatively short period of time, they are unlikely to identify all possible side effects or to ascertain the true incidence of serious drug‐related complications. Postmarketing surveillance is therefore designed to evaluate for spontaneous adverse reaction reports and identify new safety risks. The traditional approach to product safety monitoring involves collection of voluntary reports from patients and healthcare providers, a strategy called passive surveillance.^59^ Concomitantly, active surveillance, defined as regular and periodic assessment of adverse event reports from healthcare facilities and sentinel sites, uses patient registries, electronic medical record research, and prescription monitoring^59^ to provide complementary pharmacovigilance input. The integration of passive and active postmarketing surveillance permits broader epidemiological analyses of safety signals.

Several passive surveillance databases are used for drug safety monitoring in the postmarketing environment. These include VigiBase, a platform regulated by the World Health Organization; the Adverse Event Reporting System of the FDA; the Vaccine Adverse Event Reporting System; and EudraVigilance, a European Medicines Agency initiative. These databases are useful for elucidation of rare events that might be underappreciated in clinical trials, providing a window into the clinical experience of heterogenous patient cohorts who might not have been eligible for trial participation because of comorbid conditions or unfavorable performance status. The evaluation of low‐frequency longer‐term complications in high‐risk groups is therefore a key virtue of postmarketing surveillance and optimizes detection of safety events and performance of downstream epidemiological studies.

The impact of passive surveillance on cardiotoxicity monitoring is highlighted by the recent recognition of a wide spectrum of cardiovascular complications in patients treated with ibrutinib. A highly efficacious selective tyrosine kinase inhibitor, ibrutinib is a standard first‐line targeted therapeutic in patients with B‐cell lymphoproliferative neoplasms. In trials leading to its regulatory approval,^60^, ^61^, ^62^, ^63^ ibrutinib was associated with low rates of atrial fibrillation (3%–6%) and systemic bleeding. Between the years 2013 and 2017, the FDA had approved ibrutinib for patients with chronic lymphocyte leukemia, mantle cell lymphoma, Waldenström macroglobulinemia, marginal zone lymphoma, and chronic graft‐versus‐host disease, recognizing its hematologic benefits while acknowledging the potential for atrial arrhythmias. In 2018, a study of ibrutinib combinations in older adults with chronic lymphocytic leukemia demonstrated high death rates among patients enrolled in the ibrutinib arms (7% versus 1%).^64^ A subsequent VigiBase database query demonstrated that ibrutinib was associated with significantly higher reporting of supraventricular arrhythmias (reporting odds ratio, 23.1; 95% CI, 21.6–24.7; *P*<0.0001), heart failure (reporting odds ratio, 3.5; 95% CI, 3.1–3.8; *P*<0.0001), ventricular arrhythmias (reporting odds ratio, 4.7; 95% CI, 3.7–5.9; *P*<0.0001), and hypertension (reporting odds ratio, 1.7; 95% CI, 1.5–1.9; *P*<0.0001).^65^ The renewed understanding of ibrutinib and its attendant cardiovascular toxicity has helped inform individualized risk‐benefit assessment and highlighted the importance of blood pressure and arrhythmia screening, along with risk factor modification, in ibrutinib‐treated patients. It has also prompted the study and approval of second‐generation Bruton tyrosine kinase inhibitors (ie, acalabrutinib, zanubrutinib), which appear to have a milder profile of cardiovascular toxicity, although longer follow‐up is needed.

Unlike passive surveillance, active surveillance involves real‐world analyses of comprehensive databases using computerized engines that enhance timeliness and efficiency. The FDA’s Active Postmarket Risk Identification and Analysis and Sentinel System are representative tools that take advantage of advanced epidemiological methods to monitor product safety, identify and evaluate signals, and investigate risks discovered through internal and external mechanisms.^66^ With active surveillance, data are primarily derived from administrative and claims data that are generated by national health insurers and managed care organizations. Beyond pinpointing previously undescribed side effects, newer active surveillance technologies allow deep characterization of drug use in unique populations (ie, elderly, women) and assessment of drug use patterns, with an emphasis on dynamic and longitudinal reevaluation of data.^66^

## Fixing the Preclinical‐to‐Clinical Testing Disequilibrium

The hERG proarrhythmia assay provides early screening of QTc interval prolongation; however, the desired battery of biomarkers should define the risk of a wide array of interconnected cardiotoxicity phenotypes. These include myocardial dysfunction, valvular heart disease, peripheral vascular disease, stroke, myocarditis, arrythmia, and coronary artery spasm, which are prevalent drug‐related complications in oncology. For example, contraction and relaxation kinetics can be assessed using high‐speed video microscopy with motion vector analysis.^67^ In this assay, traction force microscopy and multielectrode arrays are applied to a hiPSC‐derived cardiomyocyte monolayer and cellular motion is characterized using extracellular field potential, traction force, and Ca^2+^ currents.^67^ In one study, electrical and mechanical responses to isoproterenol included an increase in the Ca^2+^ transient amplitude, upstroke, and decay, consistent with increased maximum relaxation speed and contraction speed.^67^ As illustrated by this experiment, motion kinetics in hiPSC‐derived cardiomyocytes may provide valuable information regarding electrical and mechanical events induced by pharmacologic agents. Yet the technique is limited by regional heterogeneity in contractile assessment because of nonuniform cell density and presence of noncardiac cells.

Recent advances have allowed for the development of high throughput hiPSC‐derived cardiomyocyte assays, which provide important functional output about mechanical and electrophysiologic changes. To enhance the translatability of drug‐mediated toxicity characterization to clinical settings, multiple methods are used to optimize how hiPSC‐derived cardiomyocytes recapitulate adult cardiomyocytes. These tools include hormonal influences, culture substrate adaptation, and 3‐dimensional tissue engineering, yet a gold standard protocol for hiPSC‐derived cardiomyocytes production has not been adopted.^68^

Future challenges in the study of hiPSC‐derived cardiomyocytes include refining laboratory techniques to address residual differences in morphology, contractility, metabolism, and electrophysiology. In addition, technological iterations are needed to ensure that assays evaluate the multifactorial effects of drug combinations and the impact of drugs on diseased myocardium and vasculature, as well as the biologic relationship between innate and adaptive immune system and cardiovascular processes such as myocarditis and atherosclerosis. For example, newly employed oncologic strategies use immune checkpoint inhibitor combinations with multitargeted tyrosine kinase inhibitors, raising the potential for cardiovascular toxicities that stem from blockade of upregulated compensatory mechanisms. Single‐drug evaluations are not designed to capture synergistic toxicities that result from combination therapies and may therefore underestimate the spectrum and magnitude of cardiotoxicity. To adequately quantify the clinical risks associated with anticancer drug combinations, it is essential that the integrated effects of novel polymechanism regimens on endothelial function, action potential generation and propagation, mitochondrial biogenesis, cellular contraction, and lipid metabolism are systematically studied. This is ideally performed in vitro when different drug combinations, administered concomitantly or sequentially, are evaluated for a clinical effect (ie, arrhythmia), such as with hiPSC‐derived cardiomyocyte cell assays.^69^ In addition, antibody‐drug conjugates (ie, trastuzumab emtansine, inotuzumab ozogamicin), an innovative drug delivery platform consisting of target‐specific monoclonal antibodies linked to cytotoxic molecules, should undergo dedicated cardiovascular monitoring with particular attention to inflammatory processes (ie, pericarditis, myocarditis) given their association with cytokine release syndrome and systemic inflammatory responses.

## Inclusion of Patients With Cardiovascular Disease in Oncology Trials

Although patients with cancer have a high prevalence of cardiovascular conditions, clinical trials in oncology frequently exclude participants with cardiovascular disease. In a study of 58 phase 3 breast cancer trials initiated between 1993 and 2012, heart failure, ischemic heart disease, and hypertension served as exclusion criteria in 34%, 24%, and 17% of the trials, respectively.^70^ In an analysis of 189 trials supporting 123 FDA‐approved anticancer therapies (1998–2018), 34% of the trials excluded patients with cardiovascular disease.^71^

Restrictive inclusion of patients with cardiovascular diseases poses serious challenges to the interpretation of oncology trials. First, limited generalizability undermines synthesis of high‐value clinical evidence and provides data of uncertain inference to the care of real‐world patients. This is particularly relevant in an era of an aging cancer population and longer treatment durations whereby patients are at increased risk of accumulating cardiovascular risk factors or diseases as they go through the phases of cancer treatment. In addition, recent evidence suggests that already at the time of diagnosis, patients with de novo cancers have abnormal cardiovascular imaging characteristics, as defined by cardiac magnetic resonance imaging, including smaller chamber sizes, increased strain amplitude, and systolic strain rate, and therefore various cardiovascular phenotypes will invariably exist in a given cohort of cancer patients.^72^ Second, narrow eligibility criteria compound the evaluation of drug‐drug and drug‐disease interactions, which is vital to the development of safe pharmacologic treatment practices. For example, epidermal growth factor receptor inhibitors are a cornerstone in the treatment of patients with advanced lung adenocarcinoma who harbor epidermal growth factor receptor mutations but may prolong the QT interval. The study of these medications in a biased patient population that lacks cardiovascular disease will therefore lead to underestimation of its risk for torsade de pointes. In addition, narrow inclusion limits the opportunity to examine the impact of anticancer interventions (ie, anti‐inflammatory drugs, free radical scavengers) on cardiovascular conditions. For instance, anti‐interleukin 6 monoclonal antibodies have been recently investigated as a potential treatment for renal cell carcinoma, although efficacy metrics included only oncologic measures and not cardiovascular disease end points, thus missing an opportunity to understand the compound’s potential cardioprotective effects.^73^ Finally, rare drug‐mediated cardiovascular complications may preferentially occur in at‐risk individuals who have concomitant cardiovascular diseases. This concept is known as “hidden cardiotoxicity,” whereby cardiovascular risk factors and comorbidities lower cardiac ischemic tolerance and manifest with clinically evident cardiotoxicity only in patients with preexisting ischemic myocardium or arrhythmogenic substrate, but not in healthy individuals.^74^ Studying patients with cancer who are otherwise healthy may provide false reassurance for an expanding cohort of patients who do have cardiovascular disease. This is particularly important, as many of the risk factors for cardiovascular disease and cancer, such as smoking and obesity, are shared.

**Figure 2:**
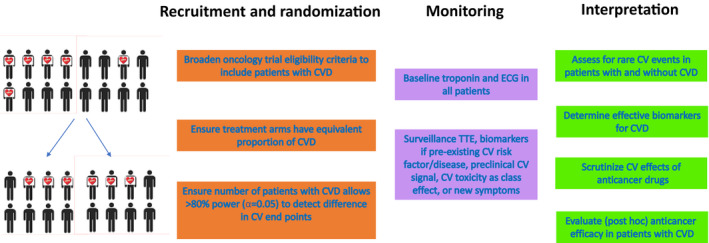
Framework for optimizing cardiovascular data analysis in oncology trials. CV indicates cardiovascular; CVD, cardiovascular disease; ECG, electrocardiography; and TTE, transthoracic echocardiography.

We maintain that oncology clinical trials should broaden eligibility criteria and routinely enroll patients with subclinical and clinical cardiovascular diseases (Figure 2). The shifting epidemiologic landscape (older patients, higher rates of cardiovascular disease, longer treatment durations, potential drug‐drug interactions, new mechanisms of cytotoxicity and immune toxicity) warrants systematic assessment of drug performance in patients with competing morbidity and mortality attributable to noncancer conditions. Quantifying benefits and harms in real‐world populations is important to create a more accurate estimate of a drug’s overall impact on clinically meaningful outcomes.^75^ Although treating cardiovascular patients with novel cancer therapies could possibly result in harm, patient consent and engagement, close clinical monitoring, and dynamic and transparent safety assessment and opt‐out options would ensure that benefits outweigh risks for the individual patient (Figure 2). Interference with trial validity can be minimized by optimizing randomization and interarm representation of patients with specific cardiovascular conditions, development of comorbidity‐adjusted monitoring protocols, and use of intention‐to‐treat analyses.

## Universal Cardiovascular Surveillance With Biomarkers and Imaging

**Figure 3:**
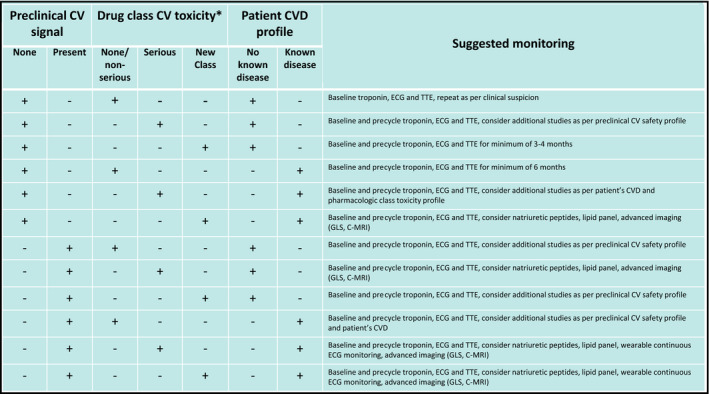
Proposed risk‐adapted protocol for cardiovascular safety monitoring in oncology clinical trials. C‐MRI indicates cardiac magnetic resonance imaging; CV, cardiovascular; CVD, cardiovascular disease; GLS, global longitudinal strain; and TTE, transthoracic echocardiography. *Drug class cardiovascular toxicity (mean incidence>0.5%): non‐serious: hypertension, low‐grade arrhythmia, pericarditis, dyslipidemia; serious: heart failure, cardiogenic shock, ischemic heart disease, high‐grade arrhythmia, myocarditis, hypotension, valvular heart disease, QTc prolongation.

Not all oncology trial participants require routine cardiovascular monitoring. Rather, a risk‐adapted strategy is suggested, whereby specific circumstances merit periodic surveillance with validated assays such as cardiac troponins and echocardiography, throughout the course of a clinical investigation. These circumstances include the combination of risk factors, presence of a preexisting cardiovascular risk factor or manifest disease, preclinical cardiotoxicity signal, drug class, and the development of new cardiorespiratory symptoms. Biomarker and imaging intervals should be devised for the individual trial and determined according to the presumed drug and cancer cohort‐specific risks. A suggested protocol is included (Figure 3). For example, it is reasonable to perform testing (biomarkers, ECG, transthoracic echocardiography) for the first 6 months in a patient with coronary artery disease participating in a study of a drug whose pharmacologic class is not associated with serious cardiovascular events and for which no concerning toxicity signals emerged in preclinical testing, and subsequently lower the frequency of testing, assuming no new symptoms or signs developed during therapy. Conversely, in the case of a patient with heart failure receiving a drug with a high incidence (5%–20%) of QTc interval prolongation or cardiac arrythmia, it might be prudent to continue monitoring beyond the first 6 months and consider additional studies, such as Holter monitoring or disposable electrode patches. Study protocols should outline recommended interventions in scenarios of asymptomatic imaging or biomarker abnormalities. This includes defining the thresholds to initiate cardioprotective agents, as well as recommended pharmacologic classes, agents, and dosages.

Finally, dual assessment of cardiovascular and oncologic outcomes will help elucidate the aggregate health effects of an investigated drug. This is particularly important given the expanding pool of pharmacotherapies that modulate inflammation and immune pathways, which extend beyond drug development in oncology to gastroenterology and rheumatology. For example, the Oral Rheumatoid Arthritis triaL is investigating the effects of tofacitinib, a small‐molecule Janus kinase inhibitor used in rheumatoid arthritis, on cardiovascular health and cancer incidence.^76^ Trial designs that allow longitudinal follow‐up for cardiovascular and cancer outcomes are strongly desired and are likely to improve the management of both diseases.

## CONCLUSIONS

**Figure 4:**
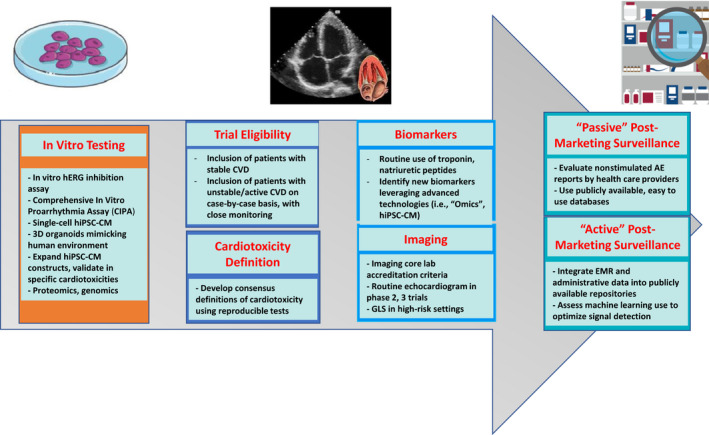
Components of cardiovascular safety assessment in the study of anticancer drugs and suggested interventions to improve early identification of clinically significant cardiotoxicity. AE indicates adverse events; CV, cardiovascular; CVD, cardiovascular disease; EF, ejection fraction; EMR, electronic medical record; hERG, human ether‐a‐go‐go–related gene; hiPSC‐CM, human induced pluripotent stem cell–derived cardiomyocytes; and GLS, global longitudinal strain.

Although substantial progress has been achieved in the clinical safety assessment of anticancer drugs, serious cardiovascular toxicities may only become evident after a large cumulative dose of a drug or its metabolites has accumulated in the heart. Alternatively, certain toxicities may be so rare that millions of individuals may be exposed before a safety signal is captured. In light of these challenging confounders, newer methods of ascertaining cardiovascular safety are desired with emphasis on early and accurate signal detection in preclinical stages, structured monitoring in clinical trial phases, and robust and active postmarketing analyses (Figure 4).

## Sources of Funding

None.

## Disclosures

Dr Neilan was supported, in part, by funding from the National Institutes of Health/National Heart, Lung, and Blood Institute (RO1HL130539, RO1HL137562, and K24HL150238), and National Institutes of Health/Harvard Center for AIDS Research (P30 AI060354). Dr Neilan has been a consultant to and received fees from H3‐Biomedicine, Abbvie, Genentech, and Amgen outside of the current work. Dr Neilan also reports consultant fees from Bristol Myers Squibb for a scientific advisory board focused on myocarditis related to immune checkpoint inhibitors and grant funding from AstraZeneca. Dr Fradley reports research grants from Medtronic and consulting fees from Abbott and AstraZeneca. Dr Bhatt discloses the following relationships: Advisory Board: Boehringer Ingelheim, Cardax, CellProthera, Cereno Scientific, Elsevier Practice Update Cardiology, Janssen, Level Ex, Medscape Cardiology, MyoKardia, NirvaMed, Novo Nordisk, PhaseBio, PLx Pharma, Regado Biosciences, and Stasys; Board of Directors: Boston VA Research Institute, Society of Cardiovascular Patient Care, TobeSoft; Chair: Inaugural Chair, American Heart Association Quality Oversight Committee; Data Monitoring Committees: Baim Institute for Clinical Research (formerly Harvard Clinical Research Institute, for the PORTICO trial [Portico Re‐sheathable Transcatheter Aortic Valve System US Investigational Device Exemption trial], funded by St. Jude Medical, now Abbott), Boston Scientific (Chair, PEITHO trial [Pulmonary Embolism Thrombolysis trial]), Cleveland Clinic (including for the ExCEED trial, funded by Edwards), Contego Medical (Chair, PERFORMANCE 2), Duke Clinical Research Institute, Mayo Clinic, Mount Sinai School of Medicine (for the ENVISAGE trial [Edoxaban Compared to Standard Care After Heart Valve Replacement Using a Catheter in Patients With Atrial Fibrillation], funded by Daiichi Sankyo), Novartis, Population Health Research Institute; Honoraria: American College of Cardiology (Senior Associate Editor, Clinical Trials and News, ACC.org; Chair, ACC Accreditation Oversight Committee), Arnold and Porter law firm (work related to Sanofi/Bristol‐Myers Squibb clopidogrel litigation), Baim Institute for Clinical Research (formerly Harvard Clinical Research Institute; RE‐DUAL PCI clinical trial steering committee funded by Boehringer Ingelheim; AEGIS‐II executive committee funded by CSL Behring), Belvoir Publications (Editor in Chief, Harvard Heart Letter), Canadian Medical and Surgical Knowledge Translation Research Group (clinical trial steering committees), Duke Clinical Research Institute (clinical trial steering committees, including for the PRONOUNCE trial [A Trial Comparing Cardiovascular Safety of Degarelix Versus Leuprolide in Patients With Advanced Prostate Cancer and Cardiovascular Disease], funded by Ferring Pharmaceuticals), HMP Global (Editor in Chief, *Journal of Invasive Cardiology*), *Journal of the American College of Cardiology* (Guest Editor; Associate Editor), K2P (Co‐Chair, interdisciplinary curriculum), Level Ex, Medtelligence/ReachMD (CME steering committees), MJH Life Sciences, Piper Sandler, Population Health Research Institute (for the COMPASS operations committee, publications committee, steering committee, and USA national co‐leader, funded by Bayer), Slack Publications (Chief Medical Editor, *Cardiology Today’s Intervention*), Society of Cardiovascular Patient Care (Secretary/Treasurer), WebMD (CME steering committees); Other: Clinical Cardiology (Deputy Editor), NCDR‐ACTION Registry Steering Committee (Chair), VA CART Research and Publications Committee (Chair); Research Funding: Abbott, Afimmune, Amarin, Amgen, AstraZeneca, Bayer, Boehringer Ingelheim, Bristol‐Myers Squibb, Cardax, CellProthera, Cereno Scientific, Chiesi, CSL Behring, Eisai, Ethicon, Ferring Pharmaceuticals, Forest Laboratories, Fractyl, Garmin, HLS Therapeutics, Idorsia, Ironwood, Ischemix, Janssen, Lexicon, Lilly, Medtronic, MyoKardia, NirvaMed, Novartis, Novo Nordisk, Owkin, Pfizer, PhaseBio, PLx Pharma, Regeneron, Roche, Sanofi, Stasys, Synaptic, The Medicines Company, and 89Bio; Royalties: Elsevier (Editor, *Cardiovascular Intervention: A Companion to Braunwald’s Heart Disease*); Site Co‐Investigator: Abbott, Biotronik, Boston Scientific, CSI, St. Jude Medical (now Abbott), Philips, and Svelte; Trustee: American College of Cardiology; Unfunded Research: FlowCo, Merck, and Takeda.
